# Divergent phenotypic response of rice accessions to transient heat stress during early seed development

**DOI:** 10.1002/pld3.196

**Published:** 2020-01-12

**Authors:** Puneet Paul, Balpreet K. Dhatt, Jaspreet Sandhu, Waseem Hussain, Larissa Irvin, Gota Morota, Paul Staswick, Harkamal Walia

**Affiliations:** ^1^ Department of Agronomy and Horticulture University of Nebraska‐Lincoln Lincoln NE USA; ^2^ Department of Animal and Poultry Sciences Virginia Polytechnic Institute and State University Blacksburg VA USA; ^3^ International Rice Research Institute Los Banos Philippines

**Keywords:** endosperm, genetic diversity, heat stress, rice, seed development, syncytial

## Abstract

Increasing global surface temperatures is posing a major food security challenge. Part of the solution to address this problem is to improve crop heat resilience, especially during grain development, along with agronomic decisions such as shift in planting time and increasing crop diversification. Rice is a major food crop consumed by more than 3 billion people. For rice, thermal sensitivity of reproductive development and grain filling is well‐documented, while knowledge concerning the impact of heat stress (HS) on early seed development is limited. Here, we aim to study the phenotypic variation in a set of diverse rice accessions for elucidating the HS response during early seed development. To explore the variation in HS sensitivity, we investigated *aus* (1), *indica* (2)*, temperate japonica* (2)*, and tropical japonica* (4) accessions for their HS (39/35°C) response during early seed development that accounts for transition of endosperm from syncytial to cellularization, which broadly corresponds to 24 and 96 hr after fertilization (HAF), respectively, in rice. The two *indica* and one of the *tropical japonica* accessions exhibited severe heat sensitivity with increased seed abortion; three *tropical japonicas* and an *aus* accession showed moderate heat tolerance, while *temperate japonicas* exhibited strong heat tolerance. The accessions exhibiting extreme heat sensitivity maintain seed size at the expense of number of fully developed mature seeds, while the accessions showing relative resilience to the transient HS maintained number of fully developed seeds but compromised on seed size, especially seed length. Further, histochemical analysis revealed that all the tested accessions have delayed endosperm cellularization upon exposure to the transient HS by 96 HAF; however, the rate of cellularization was different among the accessions. These findings were further corroborated by upregulation of cellularization‐associated marker genes in the developing seeds from the heat‐stressed samples.

## 1 INTRODUCTION

Present‐day agriculture is facing multifaceted challenges. The constant shifts in global food demands associated with exponential growth in world population, shrinking arable land, and increased frequency of extreme events need to be addressed urgently to mitigate future global food security crisis (Godfray et al., 2010; Foley et al., 2011; Röth, Paul, & Fragkostefanakis, 2016). Among the climatic variables, rising temperature is one of the most detrimental for crop yields (Porter & Gawith, 1999; Zhao et al., 2017). The average global temperature is anticipated to increase by 0.2°C per decade over the next few decades (Lobell, Schlenker, & Costa‐Roberts, 2011). In this context, a recent study estimates a 3%–8% decline in the yields of rice, wheat, and maize, for each degree‐Celsius increase in temperature (Zhao et al., 2017). These three cereal crops are a major food source worldwide; thus, a decline in yields will likely cause increasing malnutrition and socio‐economic unrest.

Rice provides 50% of the dietary caloric supply and is the primary source of income for a billion households globally, the majority of which are small landholders in Asia and Africa (Jagadish, Murt, & Quick 2015; Khush, 2005; Khush & Jena, 2009; Muthayya, Sugimoto, Montgomery, & Maberly, 2014; Van Nguyen & Ferrero, 2006). Rice grain yield, which is determined by the number of panicles per plant, number of grains per panicle, ratio of filled grains, and their weight (Sakamoto & Matsuoka, 2008; Xing & Zhang, 2010), is sensitive to high temperatures. The heat stress (HS) sensitivity of rice, particularly during reproductive development, pollen development, pollination, or anthesis, can dramatically reduce spikelet fertility (Ali et al., 2019; Arshad et al., 2017; Jagadish, Craufurd, & Wheeler, 2007; Prasad, Boote, Allen, Sheehy, & Thomas, 2006). On the other hand, HS during grain filling can negatively impact the grain quality (Sreenivasulu et al., 2015). HS exposure during early to mid‐grain filling period impairs starch biosynthesis, triggering non‐uniform filling of starch granules that lead to a chalky grain appearance (Fitzgerald & Resurreccion, 2009; Kadan, Bryant, & Miller, 2008; Lisle, Martin, & Fitzgerald, 2000). These grains are more prone to breakage during the milling process (Sreenivasulu et al., 2015). Despite our understanding of HS response of rice during vegetative, reproductive, and grain filling stages, information on impact of HS on early seed development in rice is sparse.

Endosperm is the dominant tissue and the primary determinant of seed size in the monocot crops rice, wheat, and maize (Chaudhury et al., 2001; Gao, Xu, Shen, & Wang, 2013). Postfertilization, endosperm undergoes rapid nuclear division without cytokinesis (syncytial phase). The free nuclear divisions are followed by cell wall formation (cellularization), initially around the periphery of a central vacuole and eventually filling the entire inner cavity. Afterward, endosperm development enters the grain filling phase, where it accumulates storage compounds such as starch, proteins, and lipids, thus providing a major food source worldwide (Xing & Zhang, 2010; Zuo & Li, 2014). Previous reports have shown that HS alters the timing of syncytium–cellularization transition, where moderate HS (35/30°C) accelerates the cellularization event, while severe HS (39/35°C) delays cellularization (Chen et al., 2016; Folsom, Begcy, Hao, Wang, & Walia, 2014).

Historically, the majority of rice‐producing regions prefer specific sub‐populations, for example, *indica* in South Asia, *temperate japonica* in North Asia, and *tropical japonica* in South America (Wang et al., 2016); however, with the current climate scenario, we need to explore the diverse germplasm to breed climate resilient rice. In this context, we aimed to evaluate the effect of transient HS on early seed development in a diverse set of rice accessions and determine the outcomes of this stress at seed maturity. Our subset of rice accessions represented the four sub‐populations (*aus, indica, temperate japonica,* and *tropical japonica*). Based on their response, we categorized the accessions into highly sensitive, moderately, and strongly heat tolerant. Our work provides an important component for the knowledgebase needed to utilize a diverse gene pool to develop heat‐resilient rice.

## 2 MATERIALS AND METHODS

### 2.1 Plant material and growth conditions

Nine rice accessions from Rice Diversity Panel 1 (RDP1; Zhao et al., 2011) were selected based on their relatively similar flowering time (<120 days) to ensure that the accessions flowered under similar light conditions in the glasshouse. Genetic and geographical diversity of the accessions was also considered (Table 1). Mature seeds were dehusked using a Kett TR‐130, sterilized with water and bleach (40%), and germinated in dark on half‐strength Murashige and Skoog media. After 5 days, germinated seedlings were transplanted to soil in 4‐inch square pots, and plants were grown in control glasshouse conditions: 16‐hr light and 8‐hr dark at 28 ± 1°C and 23 ± 1°C, respectively and relative humidity of 55%–60% was consistently maintained throughout the plants' life cycle (Sandhu et al., 2019).

**Table 1:** Genetic and geographical information of the rice accessions used in the current study

Accessions	GSOR Id	NSFTV Id	IRGC Id	Common name	Sub‐population	Country of origin
AUS‐1	301219	228	117673	CA 902/B/2/1	*aus*	Chad
IND‐1	301163	172	117944	Zhenshan 2	*indica*	China
IND‐2	301221	231	117753	Hunan Early Dwarf 3	*indica*	China
TEJ‐1	301110	118	117828	Oro	*temp. jap.*	Chile
TEJ‐2	301195	204	117860	Razza 77	*temp. jap.*	Italy
TRJ‐1	301141	150	117897	Sultani	*trop. jap.*	Egypt
TRJ‐2	301217	226	117762	IRAT 44	*trop. jap.*	Burkina Faso
TRJ‐3	301369	386	117839	Palmyra	*trop. jap.*	United States
TRJ‐4	301374	391	117706	Della	*trop. jap.*	United States

Note

First column refers to the names used in this study for the respective accessions. Genetic Stocks Oryza collection identification number (GSOR Id); National Science Foundation “Exploring the genetic basis of transgressive variation in rice” accessions; identification number (NSFTV Id); International Rice Genebank Collection identification number (IRGC); *temp. jap.*—*temperate japonica*; *trop. jap.*—*tropical japonica*.

### 2.2 Heat stress treatments

**Figure 1:**
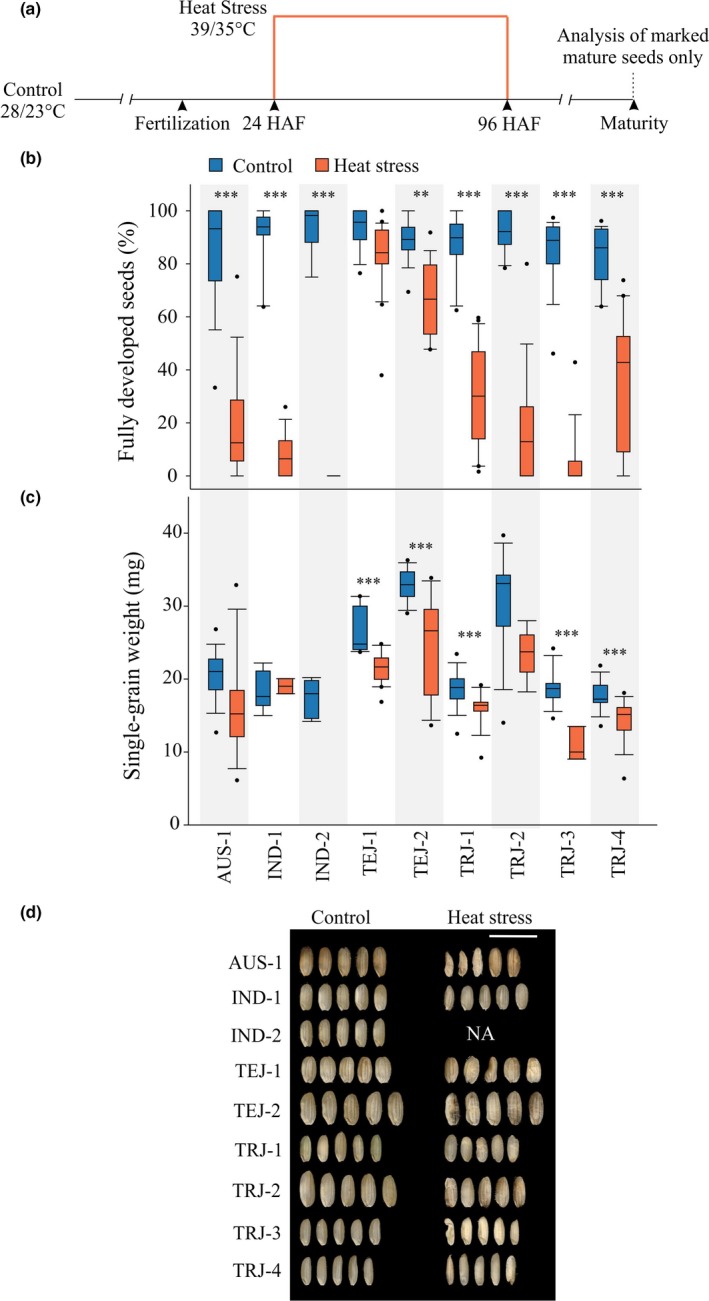
Response to post‐zygotic transient heat stress exhibits high level of phenotypic variation in seed viability among a diverse set of rice accessions. (a) Florets were marked at the time of fertilization. Twenty‐four hours after fertilization (HAF), plants were subjected to HS (39/35°C; day/night) or kept in control conditions (28/23°C; day/night). The plants were heat stressed for 3 days (i.e., until 96 HAF) and returned to control conditions. At maturity, only the marked florets (now seeds) were scored as sterile or fully developed seeds. (b) Percentage of fully developed seeds and (c) single‐grain weight from control and heat‐stressed plants. Box plots represent range and median for the same. For statistical analysis, paired *t* test was used to compare the two treatments; *n* = 700–1,000 marked seeds from 25 to 40 plants, *** indicates *p* < .001 and ** *p* < .01. (d) Representative mature seed images from control and transient heat‐stressed accessions. Images were digitally extracted and scaled for comparison (scale 1 cm). NA: not available as no mature seeds were recovered for the respective accessions

**Figure 2:**
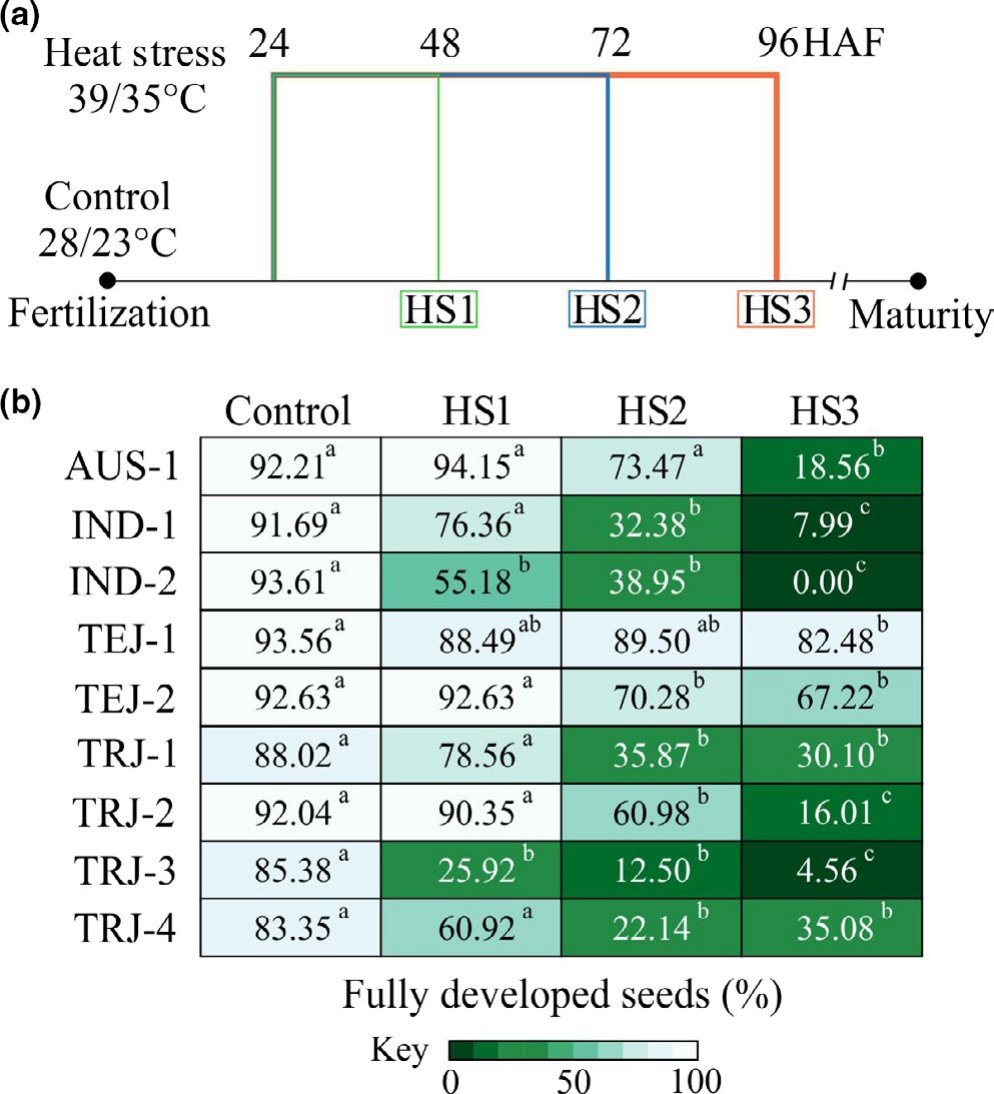
Varying duration of the heat stress provides developmental context to stress sensitivity and tolerance among the rice accessions. (a) Plants with marked florets were moved to control (28/23°C; day/night) or HS (39/35°C; day/night) after 24 hr of fertilization. Heat stress of 1 day (HS1, i.e., until 48 HAF), 2 days (HS2, i.e., until 72 HAF), or 3 days (HS3, i.e., until 96 HAF) was imposed and then relieved. Only the marked seeds harvested at maturity were considered for evaluation. (b) Heat map represents the percentage of fully developed seeds corresponding to control, HS1, HS2, and HS3 treatments. The use of white font is only to improve text visibility in the darker background. For statistical analysis, Student's *t* test was used to compare the treatments for a respective accession; *n* = 400–1,000 marked seeds from 20 to 40 plants. Mean values not sharing the same letter are significantly different (*p* < .05). HAF, hours after fertilization; C, control; HS, heat stress

Plants were grown in control conditions until flowering (Figure 1a). To track developing seeds, florets were marked at the time of fertilization. Twenty‐four hours after fertilization (HAF), plants were kept in either control conditions (16‐hr light and 8‐hr dark at 28 ± 1°C and 23 ± 1°C) or moved to a reach‐in growth chamber for heat stress (HS) treatment (16‐hr light and 8‐hr dark at 39 ± 1°C and 35 ± 1°C). The HS treatment was applied for either 1 (HS1), 2 (HS2), or 3 (HS3) days corresponding to 48, 72, and 96 HAF, respectively (Figure 2a). Afterward, plants were moved back to the control condition until maturity for evaluating the effect of HS on mature seed traits. Three independent HS experiments (in 2016, 2017, and 2018) were conducted with 10–20 plants per treatment per accession in each experiment.

### 2.3 Analysis of developing and mature seeds

To accurately assess the effect of HS on early seed development, only the florets marked at the time of fertilization were considered for downstream analysis. For mature seed phenotyping, seeds harvested at maturity were examined. Total number of fully developed and unfilled or completely sterile seeds were scored to calculate percentage of fully developed seeds. For morphometric analysis, marked mature seeds were dehusked and scanned using Epson Expression 12,000 XL scanner. The images were analyzed using *SmartGrain* (Tanabata, Shibaya, Hori, Ebana, & Yano, 2012). For mature seed analysis, 400–1,000 marked seeds from 20 to 40 plants were evaluated. For developing seed analysis, marked seeds from control or HS‐treated plants were harvested and imaged at 24, 48, 72, and 96 HAF. The images were processed using ImageJ (Abramoff, Magalhães, & Sunanda, 2004) to extract the length of developing seeds. For developing seed analysis, 15–20 marked seeds from 3 to 4 plants were evaluated.

### 2.4 Histochemical analysis

The control and HS developing seeds (72 and 96 HAF) were harvested and fixed in 1 ml of formaldehyde (2%), acetic acid (5%), and ethanol (60%; FAE solution) for 16 hr. Tissue was then washed with 70% ethanol and stored overnight at 4°C, followed by dehydration with an ethanol series (85%, 95%, and 100%) for 1 hr each at room temperature. Samples were transferred to xylene (100%) for 2 hr, followed by transfer to 500 µl of a 1:1 mixture of xylene and paraplast tablets and stored at 60°C. Finally, samples were transferred to and embedded in paraplast (Paul et al., 2020). Cross sections (10 µm) were obtained using a rotary microtome (Leica RM2125 RTS). Images from the cross sections were observed using bright‐field microscope (Leica DM‐2500). For the experiment, six to eight developing seeds for each developmental stage from two independent experiments were considered for cross sections.

### 2.5 RNA Extraction and RT‐qPCR

Total RNA was extracted from developing seeds (48 and 96 HAF) using the RNeasy Plant mini kit (Qiagen), followed by DNase treatment. One microgram of total RNA was used for cDNA synthesis using the SuperScript VILO cDNA synthesis kit (Invitrogen). The RT‐qPCR (10 µl) comprising gene‐specific primers, SYBR Green Master Mix (Bio‐Rad) and template, was conducted using LightCycler 480 Real‐Time PCR System (Roche). A ubiquitin (*UBQ5*) gene was used as an endogenous control (Jain, Nijhawan, Tyagi, & Khurana, 2006). Data were analyzed using standard methods (Livak & Schmittgen, 2001) and represented as log_2_ fold changes (Fragkostefanakis et al., 2015). For all RT‐qPCR assays, a minimum of two independent biological and three technical replicates was evaluated. The primers used to analyze the syncytial and cellularization‐associated genes are listed in Table S1.

### 2.6 Statistical analysis

Descriptive statistics and analysis of variance (ANOVA) for mature seed traits were performed in statistical software R version 3.5.3 (R Core Team 2018). Principle component analysis for traits derived from mature seeds was performed using R package *FactoMineR* and *factoextra* (Lê, Josse, Rennes, & Husson, 2008). Trait correlations between mature seed traits in control and HS treatments were performed using functions *cor.matrix()* and *corrplot()* from the corrplot R package (Wei et al., 2017). Statistical analysis used for analyzing individual experiments is explained in the figure legends.

## 3 RESULTS

### 3.1 Genotypic level heat sensitivity of fully developed mature seeds is conversely related to single‐grain weight

To understand the impact of HS on early seed development, selected rice accessions (Table 1) were subjected to transient HS for three days, that is, 24–96 hr after fertilization (HAF; Figure 1a). Only the florets marked at time of fertilization were considered for downstream analysis, which ensured accurate assessment of HS impact on early seed development. We detected genotypic differences in response to transient HS during early seed development as measured by the percentage of fully developed marked seeds (Figure 1 and Table 2). In this context, 3/9 tested accessions corresponding to *indica* (IND‐1 and 2) and a *tropical japonica* (TRJ‐3) showed drastic reduction in number of fully developed seeds (Figure 1b). One *indica* accession (IND‐2) showed complete sterility under HS treatment, as we did not recover any developed mature seed, so the respective accession was not part of the downstream mature seed measurements (Figure 1b and c). On the other hand, *aus* (AUS‐1), one of the *temperate japonicas* (TEJ‐2), and three *tropical japonicas* (TRJ‐1, −2, and −4) also showed significant reductions; however, reductions were relatively less severe (Figure 1b). One of the *temperate japonicas* (TEJ‐1) exhibited strong resilience to transient HS, as number of fully developed seeds recovered in the heat‐stressed plants was comparable to the respective control plants (Figure 1b). We also measured seed parameters for the seeds that did fully develop for each accession. The *indica* accession that showed a steep decline for fully developed seeds (IND‐1) was not affected in single‐grain weight for the fully developed seeds at maturity. Contrarily, the four accessions maintaining the relatively higher mature seed numbers under stress (TEJ‐1, TEJ‐2, TRJ‐1, and TRJ‐4) showed a significant reduction with respect to single‐grain weight (Figure 1c).

**Table 2:** Analysis of variance of the nine accessions

Sources of variation	DF	*F*‐value						
		FDS	SGW	Area	Perimeter	Length	Width	L/W
Accession	8	2.64*	13.94^$^	29.87^$^	3.84*	2.92*	30.19^$^	5.43^$^
Treatment (T)	1	90.74^^^	21.96^$^	17.24^$^	0.14	5.74*	9.17^$^	0.27
Environment (E)	2	0.15	0.40	5.27*	11.88^^^	7.05^^^	3.10	1.67
T × G	8	1.94	2.65	2.05	2.30	1.70	0.54	0.53
E × G	16	0.30	1.088	1.30	1.94	1.34	0.82	0.90
Residuals	18							

Note

DF represents degrees of freedom; FDS, fully developed seeds (%); SGW, single‐grain weight; L/W, length/width.

*^,^,$^Significant level at 5%, 1%, and 0.1% level, respectively.

Given the phenotypic variability for the fully developed seeds (Figure 1d), we next dissected the impact of HS on early developing seeds in a temporal context by imposing HS for either 1 (HS1; 24 to 48 HAF), 2 (HS2; 24 to 72 HAF), or 3 (HS3; 24 to 96 HAF) days (Figure 2a). In general, the severity with respect to fully developed seeds increased with increase in duration of the HS (HS3 > HS2 > HS1 > Control; Figure 2b). Interestingly, the *aus* accession (AUS‐1), which showed a significant decrease in fully developed seeds on exposure to three days of HS (Figure 1b), was not significantly affected after 1 and 2 days of HS (HS1 and HS2, respectively; Figure 2b). In contrast, the *indica* accession (IND‐2) that exhibited complete sterility following 3 days of stress (Figure 1b) also showed a sharp decline in fully developed seeds after only one day of HS (HS1) treatment (Figure 2b). A similar trend was observed for one of the *tropical japonica* accessions (TRJ‐3), while the others (TRJ‐1, TRJ‐2, and TRJ‐4) exhibited a decrease in fully developed seeds after two days of HS (HS2) treatment (Figure 2b). The *temperate japonica* accession TEJ‐1 did not show an inflection point for the respective HS treatments, maintaining near control level of mature seeds throughout (Figure 2b). The other *temperate japonica* accession TEJ‐2 had fewer fully developed seeds under HS2 treatment but was not further affected after an additional day of stress (HS3; Figure 2b).

### 3.2 Heat stress penalizes seed length of the relatively resilient accessions

**Figure 3:**
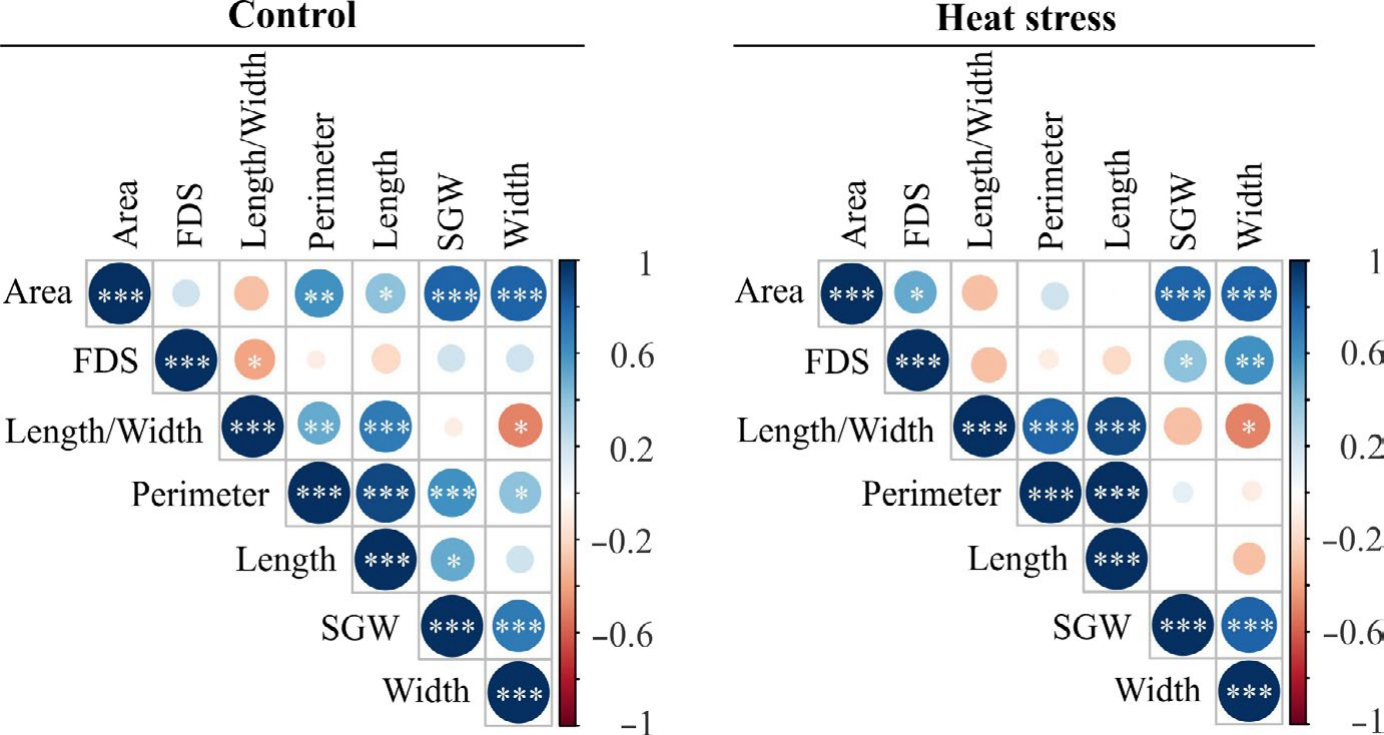
Correlation of mature seed traits. Pearson's correlation of the mature seed (only marked) traits derived from control and three days of heat stress treatment (until 96 hr after fertilization) during early seed development. *** denotes *p* < .001, **p < .01, *p < .05. FDS, percentage of fully developed seeds; SGW, single‐grain weight

To elucidate the impact of the three‐day HS during early seed development on seed size, morphometric measurements on fully developed control and heat‐stressed seeds were performed. In this context, area, perimeter, length, width, and length‐to‐width ratio of the individual seeds were evaluated. A significant difference between control and HS treatments was observed for all measured parameters for certain accessions except for length‐to‐width ratio, indicating the prevalent treatment effect (Table 3). Interestingly, both *temperate japonica* accessions (TEJ‐1 and TEJ‐2) showed a significant reduction in seed length under HS. Further, seed area and width were also reduced for TEJ‐2, while these traits were significantly heat‐tolerant for TEJ‐1 (Table 3). Among the *tropical japonica* accessions, only TRJ‐2 showed a significant reduction in seed area, length, width, and perimeter. Another *tropical japonica* accession, TRJ‐3, showed marginal reduction in seed width under HS. It is notable that the *indica* accession IND‐1 maintained seed size parameters, while the *aus* accession AUS‐1 showed reduction only in seed area (Table 3). A significant positive correlation for several examined mature seed traits was detected for both control and HS treatments. Most of these were common to both treatments, indicating possible involvement of the same genetic loci in controlling these traits or perhaps that the respective traits are under pleiotropic influence (Figure 3). As expected, seed area is significantly (*p* < .001) correlated with perimeter, length, width, and single‐grain weight under control conditions, suggesting the inter‐dependency of the analyzed traits; however, area was significantly (*p* < .001) correlated only with seed width and single‐grain weight under HS (Figure 3). Likewise, we did not detect a significant positive correlation of fully developed seeds with other traits under control, indicating that the respective trait is independent. A significant correlation was detected for single‐grain weight with seed width under control as well as HS treatments (Figure 3). Single‐grain weight was weakly correlated with seed length under control, while no correlation existed between the respective traits under HS (Figure 3).

**Table 3:** Morphometric analysis of mature seeds.

Accession	Area (mm^2^)	Perimeter (mm)	Length (mm)	Width (mm)	Length/Width
AUS‐1 Control	11.48 ± 1.0^^^	15.49 ± 1.6	6.13 ± 0.6	2.50 ± 0.1	2.56 ± 0.4
AUS‐1 HS	9.45 ± 1.9	14.53 ± 2.0	5.68 ± 0.8	2.33 ± 0.2	2.71 ± 0.8
IND‐1 Control	12.38 ± 0.7	15.08 ± 0.4	5.74 ± 0.1	2.78 ± 0.1	2.16 ± 0.2
IND‐1 HS	11.69 ± 1.3	16.70 ± 3.9	6.40 ± 1.7	2.68 ± 0.2	3.52 ± 2.9
IND‐2 Control	12.68 ± 0.5	15.17 ± 0.4	5.80 ± 0.1	2.79 ± 0.2	2.12 ± 0.2
IND‐2 HS	NA	NA	NA	NA	NA
TEJ‐1 Control	14.28 ± 0.4	15.64 ± 0.4	5.72 ± 0.1	3.22 ± 0.1	1.80 ± 0.1
TEJ‐1 HS	13.64 ± 0.8	15.39 ± 0.5	5.56 ± 0.2*	3.15 ± 0.1	1.78 ± 0.1
TEJ‐2 Control	17.18 ± 0.9	17.87 ± 1.15	6.86 ± 0.3	3.26 ± 0.1	2.19 ± 0.4
TEJ‐2 HS	15.23 ± 2.2^^^	17.05 ± 0.9	6.50 ± 0.4*	3.02 ± 0.3^^^	2.22 ± 0.2
TRJ‐1 Control	11.64 ± 0.8	14.69 ± 0.6	5.77 ± 0.2	2.58 ± 0.1	2.25 ± 0.1
TRJ‐1 HS	11.00 ± 1.8	14.31 ± 0.8	5.53 ± 0.3*	2.57 ± 0.3	2.18 ± 0.2
TRJ‐2 Control	17.40 ± 0.7	18.10 ± 0.5	7.10 ± 0.2	3.10 ± 0.1	2.3 ± 0.1
TRJ‐2 HS	13.90 ± 2.3^$^	16.00 ± 1.6^$^	6.14 ± 0.8^$^	2.90 ± 0.3*	2.10 ± 0.3
TRJ‐3 Control	12.00 ± 0.6	15.10 ± 0.6	5.93 ± 2.6	2.61 ± 0.1	2.33 ± 0.1
TRJ‐3 HS	11.10 ± 0.7	17.00 ± 2.7	6.80 ± 1.5	2.47 ± 0.2*	3.8 ± 2.3
TRJ‐4 Control	11.80 ± 1.0	16.00 ± 0.9	6.61 ± 0.4	2.22 ± 0.1	3.0 ± 0.11
TRJ‐4 HS	11..80 ± 0.3	15.94 ± 0.3	6.50 ± 0.1	2.21 ± 0.1	2.90 ± 0.1

Average morphometric measurements from mature seeds of control plants and heat stressed (HS) plants. *n* = 700–1000 marked seeds from 25 to 40 plants. Paired *t*‐test was used for statistical analyses (Control vs HS for the respective accession). NA not available. ± indicate standard deviation, ^$^Indicates *p*‐value <.001, ^^^.01, *.05.

### 3.3 Principle component analysis

**Figure 4:**
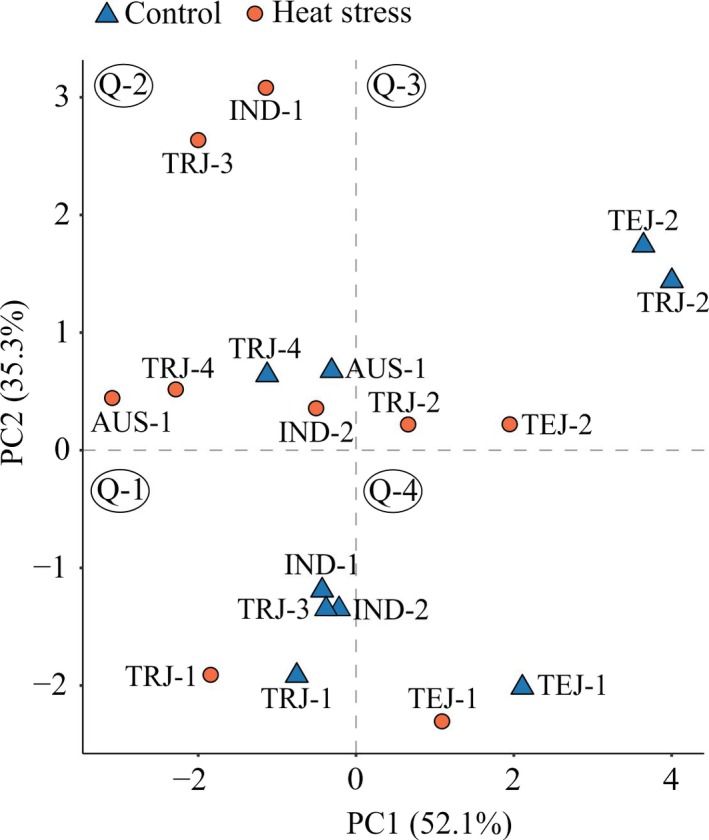
Principal component analysis (PCA) of mature seed traits analyzed from the nine rice accessions. Mature seed traits (only marked seeds) from plants subjected to control and three days of heat stress treatment (24–96 hr after fertilization) during early seed development were considered for PCA. Blue triangle and orange circle represent control and heat stress treatments, respectively, for the different rice accessions. Q‐1/2/3/4 indicates the respective quadrant

To assess phenotypic diversity among accessions under control and HS treatments, we performed principle component analysis (PCA; Figure 4) using all mature seed traits. The first two principle components PCs (1 and 2) explained 87% of total variation. PCA effectively explained the variation in the severely susceptible accessions (IND‐1, TRJ‐3, IND‐2) due to HS. Also, these accessions were clustered together under control, first quadrant, Q‐1, and HS (Q‐2; Figure 4). On the other hand, control and HS for TRJ‐1 (Q‐1), TRJ‐4 and AUS‐1 (Q‐2), TEJ‐2 and TRJ‐2 (Q‐3), and TEJ‐1 (Q‐4) exhibited relatively closer associations (Figure 4). PCA provided a broader context for phenotypic diversity by showing that the sensitive accessions exhibited distinct responses to control and HS treatments, while the relatively tolerant accessions showed comparable phenotypes in the two treatments.

### 3.4 Resilient accessions sense heat stress prior to the sensitive accessions

**Figure 5:**
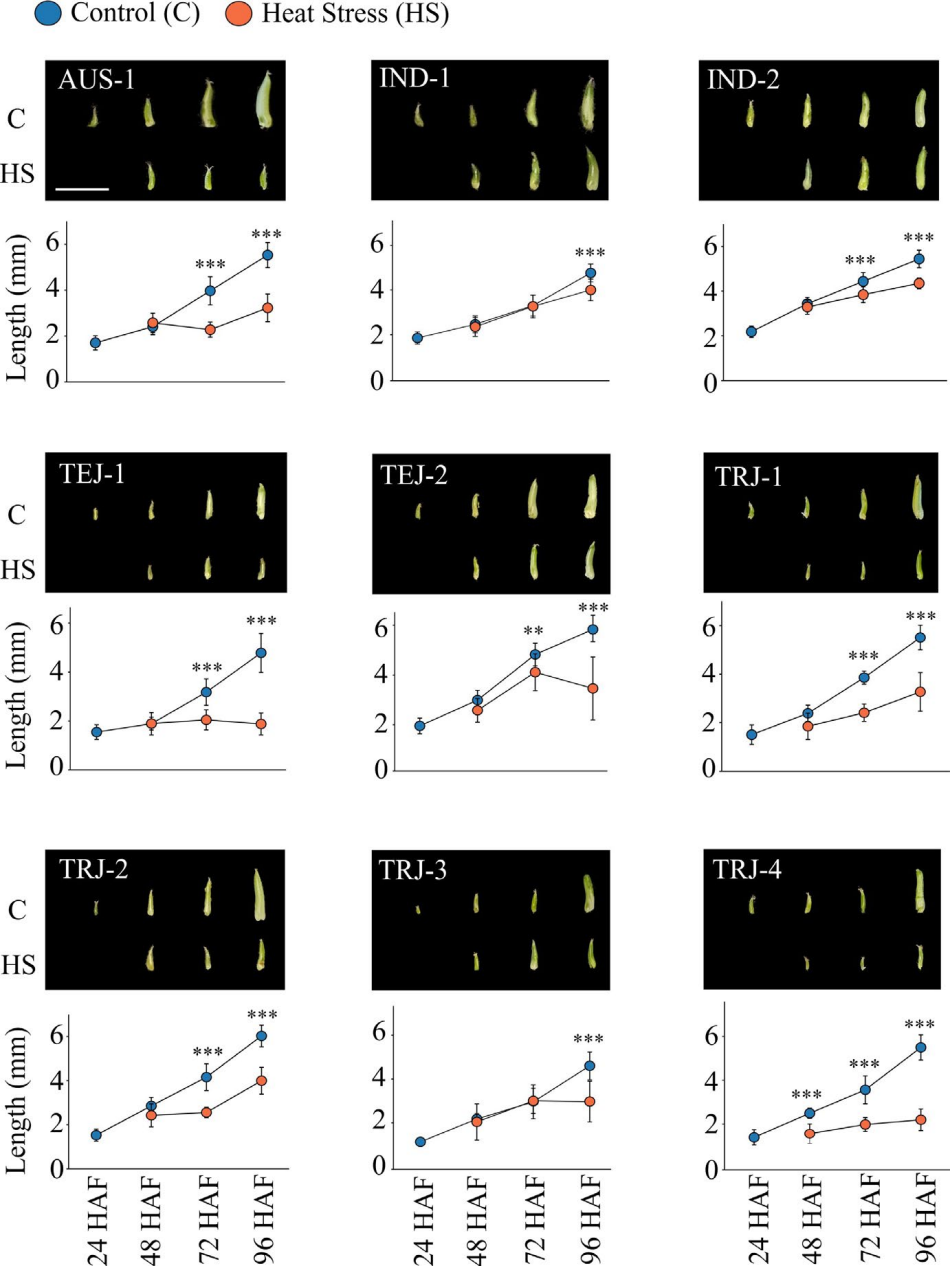
Effect of transient heat stress on length of developing young seeds. Plants with marked florets at the time of fertilization were subjected to control or HS treatment. The HS treatment, initiated 24 HAF, was for either 1 day (24–48 HAF), 2 days (24–72 HAF), or 3 days (24–96 HAF). The marked developing seeds from control and heat‐stressed plants were harvested at 48, 72, and 96 HAF. Seed length was measured using ImageJ. Representative developing seeds from the respective time‐point, treatment, and accessions are shown. Images were digitally extracted and scaled for comparison (scale 1 cm). For statistical analysis, paired *t* test was used to compare developing seed lengths from control and HS treatments for a given time‐point; *n* = 15–20 marked developing seeds, *** indicates *p* < .001 and **p < .01. HAF, hours after fertilization

Because we observed differences in seed length at maturity for four of the relatively heat‐tolerant accessions (TEJ‐1, TEJ‐2, TRJ‐1, and TRJ‐2), we next aimed to capture the genotypic differences in growth of the developing seeds exposed to control and HS treatments. Like mature seed phenotypes, varied responses among accessions were observed. From the analysis, 7/9 accessions exhibited a significant variation in the developing seed length by 72 HAF, whereas an *indica* (IND‐1) and a *temperate japonica* (TRJ‐3) showed significant changes only by 96 HAF (Figure 5). However, one of the *tropical japonica* (TRJ‐4) started showing HS effect from 48 HAF, that is, 24 hr after the HS treatment. These results suggest that developing grain response in context of grain length is predictive of lengths at maturity (Table 3) and measuring the grain length increase daily provides further insight into genotypic differences to HSR (Figure 5).

### 3.5 Histochemical analysis reveals differential rate of endosperm cellularization

**Figure 6:**
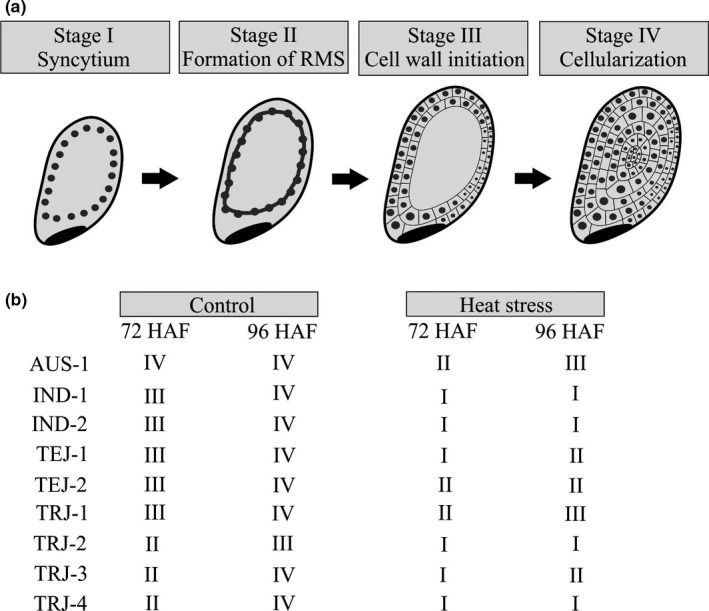
Transient heat stress during early seed development delays endosperm cellularization. (a) Pictorial representation depicting different stages of rice endosperm from syncytial to cellularization. (b) Based on the stages defined in (a), progression of endosperm development (at 72 and 96 HAF) under control and heat stress in the nine rice accessions. This assessment is based on representative cross‐section images of developing seed (Figure S2). rms, radial microtubule system; HAF, hours after fertilization

The observed differences in seed morphology at maturity and length of developing seed prompted us to explore potential genotypic variation in the cellular biology of early seed development under heat stress. Previous reports have shown that the developing endosperm is at the syncytial stage at or around 48 HAF and completes cellularization around 96 HAF in a model rice variety, Kitaake (Chen et al., 2016; Folsom et al., 2014). We observed that the cellularization timing was affected not only by HS, but also varied among accessions  even under control conditions (Figure 6 and Figure S2). For instance, the *aus* accession (AUS‐1) completed endosperm cellularization by 72 HAF under control conditions, whereas all other accessions were either showing the radial microtubule system (RMS; *tropical japonicas*: TRJ‐2, TRJ‐3, and TRJ‐4) or were initiating cell wall formation (*indicas*: IND‐1 and IND‐2, *temperate japonicas*: TEJ‐1 and TEJ‐2) by 72 HAF. Except for one of the *temperate japonicas* (TRJ‐2), all other accessions completed endosperm cellularization by 96 HAF in the control conditions (Figure 6 and Figure S2). Under the impact of transient HS, endosperm cellularization was delayed in all the tested accessions. The RMS, which precedes cell wall formation, was initiated in AUS‐1, TEJ‐2, and TRJ‐1 by 72 HAF in the HS treatment, while TEJ‐1 and TRJ‐3 exhibited RMS by 96 HAF in the HS‐treated samples (Figure 6 and Figure S2). We did not detect RMS at either 72 or 96 HAF in the heat‐stressed samples for the two *indica* accessions, IND‐1 and IND‐2 (Figure 6 and Figure S2). A similar pattern was detected for the two *tropical japonicas* (TRJ‐2 and TRJ‐4; Figure 6 and Figure S2).

### 3.6 Gene expression of syncytial‐ and cellularization‐specific marker genes corroborates the histochemical analysis

**Figure 7:**
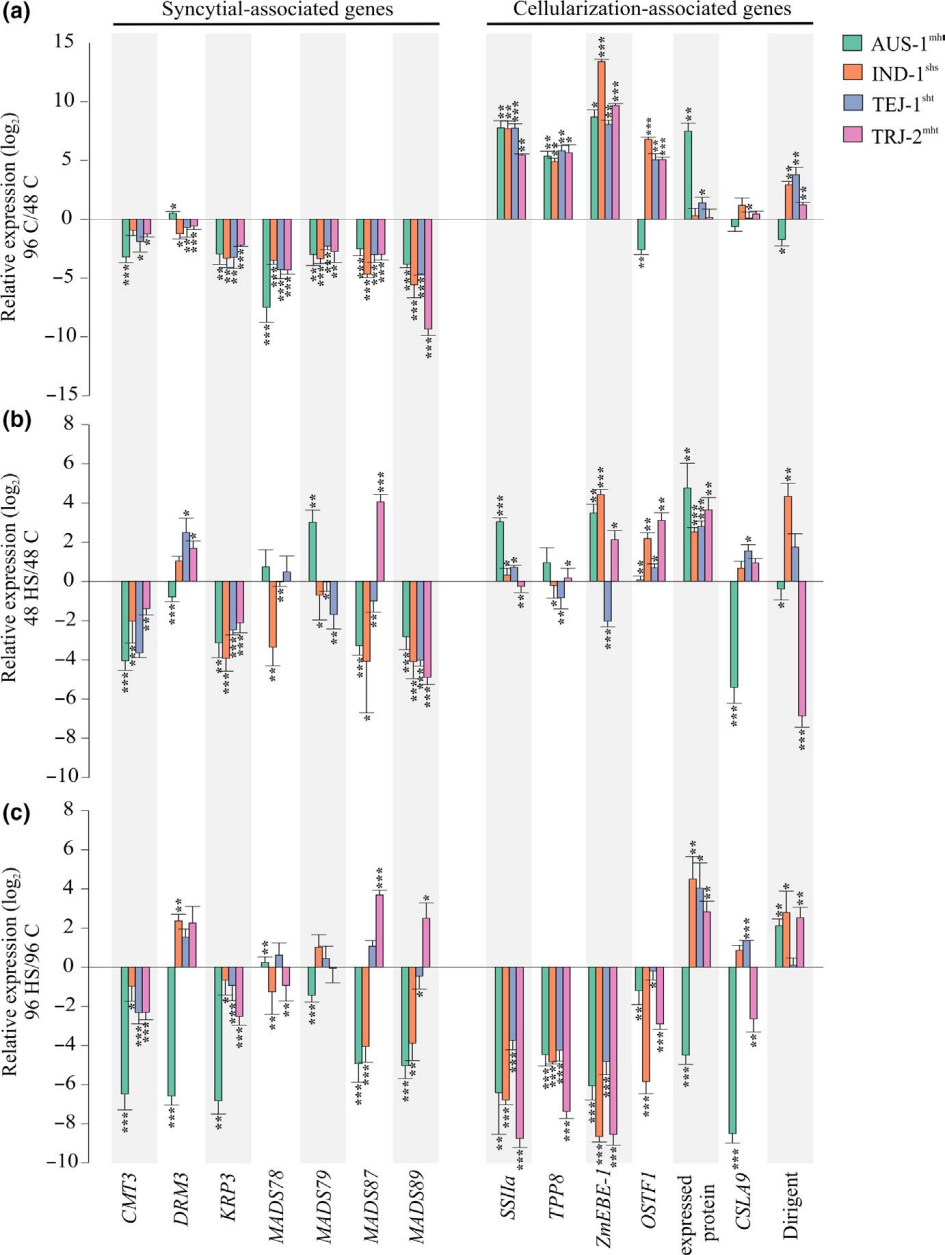
Transient heat stress alters the expression of syncytial and cellularization marker genes for early seed development. Gene expression of selected marker genes for syncytial and cellularization events during early seed development on four accessions representing different sub‐populations: *aus* (AUS‐1^mht^), *indica* (IND‐1^shs^), *temperate japonica* (TEJ‐1^sht^), and *tropical* (TRJ‐2^mht^) *japonica*. shs, mht, and sht in the superscript refer to severely heat sensitive, moderately heat tolerant, and strongly heat tolerant. (a) RT‐qPCR analysis representing the expression of the selected marker genes at 96 HAF relative to 48 HAF under control conditions. (b and c) RT‐qPCR analysis representing the expression of selected markers under stress relative to the respective controls at (b) 48 and (c) 96 HAF. For statistics, paired *t* test was used to compare relative expression levels for each gene in the respective accession. Error bars indicate standard deviation. *** indicates *p* < .001 and **p < .01. HAF, hours after fertilization; C, control; HS, heat stress

The transition of endosperm from the syncytial phase to cellularization is a major event during seed development as it defines the extent of nuclear proliferation and thereby determines final seed size (Brown, Lemmon, & Olsen, 1996; Li, Xu, Duan, & Li, 2018; Olsen, 2004). The event is finely orchestrated at the genetic and epigenetic levels (Folsom et al., 2014). *CMT3* (Folsom et al., 2014; Lindroth et al., 2001), *DRM3* (Sharma et al., 2009), *KRP3* (Mizutani, Naganuma, Tsutsumi, & Saitoh, 2010), and several type‐I MADS‐box transcription factors, *MADS78*, *MADS* *79*, *MADS* *87*, and *MADS* *89* (Chen et al., 2016; Folsom et al., 2014; Kang, Steffen, Portereiko, Lloyd, & Drews, 2008; Paul et al., 2020; Steffen, Kang, Portereiko, Lloyd, & Drews, 2008), show expression specific to the syncytial phase, and decline with the progression of cellularization. Thus, we referred these as syncytial‐associated genes (Figure 7). On the other hand, genes that are upregulated with the progression of endosperm cellularization relative to the syncytial phase, for example, *OSTF1* (Yang, Chung, Tu, & Leu, 2002), *SSIIa* (Folsom et al., 2014), *ZmEBE‐1* (French, Abu‐Zaitoon, Uddin, Bennett, & Nonhebel, 2014), and others reported by Chen *et al* (Chen et al., 2016), are referred to as cellularization‐associated (Figure 7). For these syncytial and cellularization marker genes, RT‐qPCR analysis on developing seeds (48 and 96 HAF) was performed for 4 accessions: AUS‐1, IND‐1, TEJ‐1, and TRJ‐2 representing each of the sub‐populations, *aus*, *indica*, *temperate, and tropical japonicas*, respectively. Developing seed samples at 48 and 96 HAF from control and heat‐stressed samples were chosen because they reflected syncytial and cellularization stages for most of the tested accessions (Figure 6). The primary objective of the gene expression analysis was to validate marker gene expression at 48 and 96 HAF under control conditions (Figure 6). As expected, we observed down‐ and upregulation of syncytial‐ and cellularization‐associated genes, respectively, by 96 HAF relative to 48 HAF under control conditions (Figure 7a). However, the *aus* accession (AUS‐1) showed exceptions, as *DRM3* corresponding to syncytial‐associated genes showed upregulation, while two cellularization‐associated genes (*OSTF1* and dirigent) were downregulated by 96 HAF (Figure 7a). Next, we examined the expression of the selected genes at 48 HAF under HS relative to 48 HAF control (Figure 7b). Three syncytial‐associated genes (*CMT3*, *KRP3*, and *MADS89*) were downregulated in all the tested accession for the heat‐stressed samples from 48 HAF, wherein *DRM3* and *MADS79* were significantly upregulated in at least one of the accessions (Figure 7b). Two of the cellularization‐associated genes (expressed protein: *LOC_Os05g34510* and *OSTF1*) were upregulated in all the tested accession, while other cellularization‐associated genes (*SSIIa, TPP8, ZmEBE‐1, CSLA9*, and dirigent) were downregulated in at least one of the tested accessions for the heat‐stressed samples from 48 HAF (Figure 7b). We also examined the expression of these genes at 96 HAF under HS relative to 96 HAF control (Figure 7c). In this context, 5/7 syncytial‐associated (except *CMT3* and *KRP3*) and 6/7 cellularization‐associated (except dirigent) genes were up‐ and downregulated, respectively, in at least one of the tested accessions (Figure 7c). Taken together, the gene expression level responses support the observation of developmental disruption in initiation and/or progression of endosperm cellularization under HS (Figure 6), as four of the cellularization‐specific marker genes (*SSIIa, TPP8, ZmEBE‐1,* and *OSTF1*) were downregulated in all the four tested accessions at 96 HAF in the heat‐stressed samples relative to the 96 HAF control samples (Figure 7c). While cellularization‐associated marker gene response to HS provides a clear case for endosperm development, there are no clear expression level responses that enable broad prediction of individual seed‐level tolerance or sensitivity to HS imposed in this study.

## 4 DISCUSSION

The global mean temperature rise over the last century has negatively influenced crop yields (Battisti & Science, 2009; Challinor et al., 2014; Gourdji, Sibley, & Lobell, 2013; Lobell et al., 2011; Zhao et al., 2017). The situation is expected to worsen given the predicted increase in global temperatures (Gourdji et al., 2013; Zhao et al., 2017). Extensive work has been carried out regarding the impact of HS on different phases of plant development (Bokszczanin & Fragkostefanakis, 2013; Cheng, Sakai, Yagi, & Hasegawa, 2009; Hasanuzzaman, Nahar, Alam, Roychowdhury, & Fujita, 2013; Jagadish et al., 2015; Prasad, Bheemanahalli, & Jagadish, 2017). Both reproductive development and grain filling phases in rice have been documented as sensitive to environmental perturbations (Prasad et al., 2006; Jagadish et al., 2007; Prasad et al., 2008; Jagadish et al., 2010; Coast, Ellis, Murdoch, Quiñones, & Jagadish, 2015; Arshad et al., 2017; Ali et al., 2019; Dhatt et al., 2019). However, genotypic variation in rice germplasm for heat stress responses during early seed development remains largely unexplored. In the present study, we systematically investigated the impact of HS on the early seed development that corresponds to the transition phase of endosperm from syncytial to cellularized. The rate and timing of this transition is one of the main drivers for determining final seed size (Brown et al., 1996; Li et al., 2018; Olsen, 2004). Here, we imposed transient HS (39/35°C) during early seed development (24–96 hr after fertilization; HAF) on nine rice accessions corresponding to different sub‐populations (Table 1, Figures 1 and 2). Given the HS sensitivity of pollination and/or fertilization (Zinn, Tunc‐Ozdemir, & Harper, 2010), HS was initiated 24 HAF so that it did not interfere with either event. Furthermore, we only considered seeds that were marked at the time of fertilization. This approach helped us to (i) avoid overestimation of final yield penalty caused by HS and (ii) precisely track the early seed developmental stages with respect to syncytial and endosperm cellularization and their response to HS. Thus, our analysis does not necessarily reflect whole plant yield‐level sensitivity or tolerance but rather provides a comprehensive and explicit view of the HS impact on early seed developmental stages. This approach is useful for future work that aims to identify the genetic basis of variation with respect to heat tolerance in the rice germplasm. Having developmental and temporal context will enhance prioritization of genes that increase resilience to transient heat stress during early seed development.

Heat stress response of the nine rice accessions varied widely. The two *indica* accessions, IND‐1^shs^ and IND‐2^shs^ (“shs” in the superscript refers to severely heat sensitive), and one of the *tropical japonica* accessions (TRJ‐3^shs^) showed similar heat stress response with respect to proportion of fully developed seeds (Figures 1 and 2). Also, these accessions showed clear separation of the mature seed traits derived from the two treatments, control (Q‐1) and HS (Q‐2), depicting an overall sensitivity of these accessions (Figure 4). On the other hand, *aus* (AUS‐1), two *temperate japonicas* (TEJ‐1 and TEJ‐2), and three *tropical japonicas* (TRJ‐1, TRJ‐2, and TRJ‐4) showed relatively stronger association between the control and HS treatments (Figure 4). However, because of the limited number of accessions evaluated, we cannot generalize that tolerance or sensitivity to HS during early seed development is related to place of origin or sub‐population of the accessions. Further, based on the heat stress response, the relatively tolerant accessions can be sub‐categorized as moderately and strongly heat tolerant (Figure 2b). The four accessions, AUS‐1^mht^, TRJ‐1^mht^, TRJ‐2^mht^, and TRJ‐4^mht^ (“mht” in the superscript refers to moderately heat tolerant), showed drastic reduction in fully developed seeds by either two or three days of HS (HS2 or HS3, respectively) treatment, whereas TEJ‐1^sht^ and TEJ‐2^sht^ (“sht” in the superscript refers to the strongly heat tolerant) showed mild reductions in fully developed seeds in response to HS2 or HS3 (Figure 2b). Moreover, 4/6 tolerant accessions showed a significant reduction in the mature seed lengths on exposure to three days of HS (Table 3). These reductions are possible repercussions of the significant decline in the developing seed lengths as early as 72 HAF for all the relatively tolerant accessions (Figure 5). Contrarily, the sensitive accessions did not show any reduction with respect to mature seed length; however, single‐grain weight and seed width were significantly compromised for TRJ‐3^shs^ (Table 3). Likewise, developing seed lengths for 2/3 relatively sensitive accessions (IND‐1^shs^ and TRJ‐3^shs^) did not show any alteration until 96 HAF in the HS‐treated samples (Figure 5). Thus, we hypothesize that the heat‐tolerant and the sensitive accessions (defined based on the individual seed level analysis) follow two different HSR mechanisms: the tolerant accessions, on exposure to HS, invest in maintaining the number of fully developed seeds but compromise seed size, whereas the sensitive accessions maintain mature seed size at the expense of the number of fully developed seeds. It is pertinent to state that the whole plant tolerance or sensitive response (as measured by long‐term heat stress and its impact on seed weight per plant) may be different from how sensitivity and tolerance to the imposed HS is defined in current study.

Furthermore, histochemical investigation of the accessions during early seed developmental phases revealed that all three sensitive accessions either possessed radial microtubule systems (RMS; TRJ‐3^shs^) or initiated the process of endosperm cellularization (IND‐1^shs^ and IND‐2^shs^) by 72 HAF and completed cellularization by 96 HAF under control conditions (Figure 6). The RMS defines the nuclear‐cytoplasmic domains, thus facilitating the process of endosperm cellularization (Olsen, 2004; Sabelli & Larkins, 2009). In contrast, under HS conditions, the sensitive accessions did not possess RMS by 72 HAF, suggesting substantial delay in the cellularization process (Figure 6 and Figure S2). On the other hand, 4/6 relatively tolerant accessions (AUS‐1^mht^, TRJ‐1^mht^, TEJ‐1^sht^, and TEJ‐2^sht^) either possessed RMS or commenced the cell wall formation procedure under HS by 96 HAF (Figure 6 and Figure S2). The difference in the rate of cellularization in the different accessions might be correlated with their adaptation strategies to HS. Gene expression analysis further validated the findings from the histochemical analysis. The syncytial‐specific marker genes were downregulated, while the cellularization‐specific marker genes were upregulated at 96 HAF in the control samples (Figure 7a). This observation is in accordance with the developing seed cross sections as a majority (8/9; except TRJ‐2^mht^) of the tested accessions underwent complete endosperm cellularization by 96 HAF (Figure 6 and Figure S2). Similarly, under HS conditions at 96 HAF, 5/7 syncytial‐associated and 6/7 cellularization‐associated genes were up‐ and downregulated, respectively (Figure 7c). These results align well with the histochemical findings where all the tested accessions show delay in endosperm cellularization upon exposure to HS (Figure 6). However, further investigation of gene expression in context of the rate of cellularization will require testing of marker genes corresponding to RMS or cell wall initiation.

In summary, this study provides detailed insight into the divergence of heat stress response mechanisms of a subset of rice accessions on exposure to the transient HS during early seed development. This work also emphasizes the challenges in developing a simple sampling approach that would account for these differences in developmental transitions and impact of HS in a duration‐dependent manner across accessions. Further validation of the sensitive and tolerant accessions through ‐*omic* analysis will be helpful in molecular dissection. This work will also be useful in developing future phenotyping strategies for large‐scale screening and mining allelic variants for heat resilience rice.

## CONFLICT OF INTEREST

The authors declare no conflicts of interest.

## AUTHOR CONTRIBUTIONS

PP and HW conceived and designed the experiments. PP performed all the heat stress experiments. BKD/JS/LI assisted in collection of the developing seed tissue for the downstream analysis. BKD performed histochemical analysis and RT‐qPCRs. WH and GM performed the statistical analysis. PP analyzed the results from all the experiments and wrote the manuscript. PS critically reviewed and analyzed the work. All authors read and approved the manuscript.
