# Supplementary figures and images for: Divergent phenotypic response of rice accessions to transient heat stress during early seed development
Source: Plant Direct. 2020 Jan 12;4(1):e00196. doi: 10.1002/pld3.196 (PMC6955394; doi:10.1002/pld3.196)

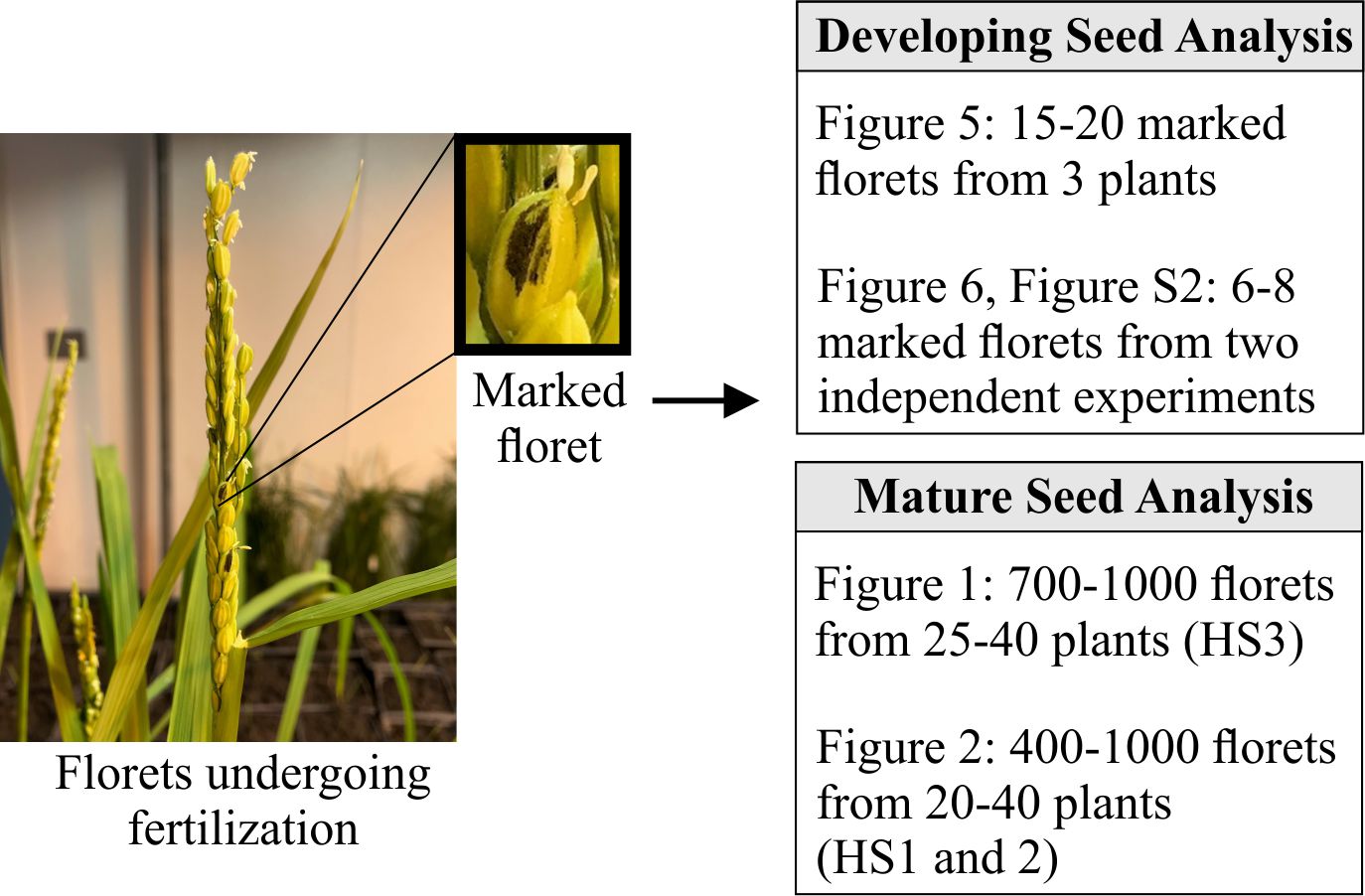

Supplement: Supplementary file 1 [file PLD3-4-e00196-s001.jpg]

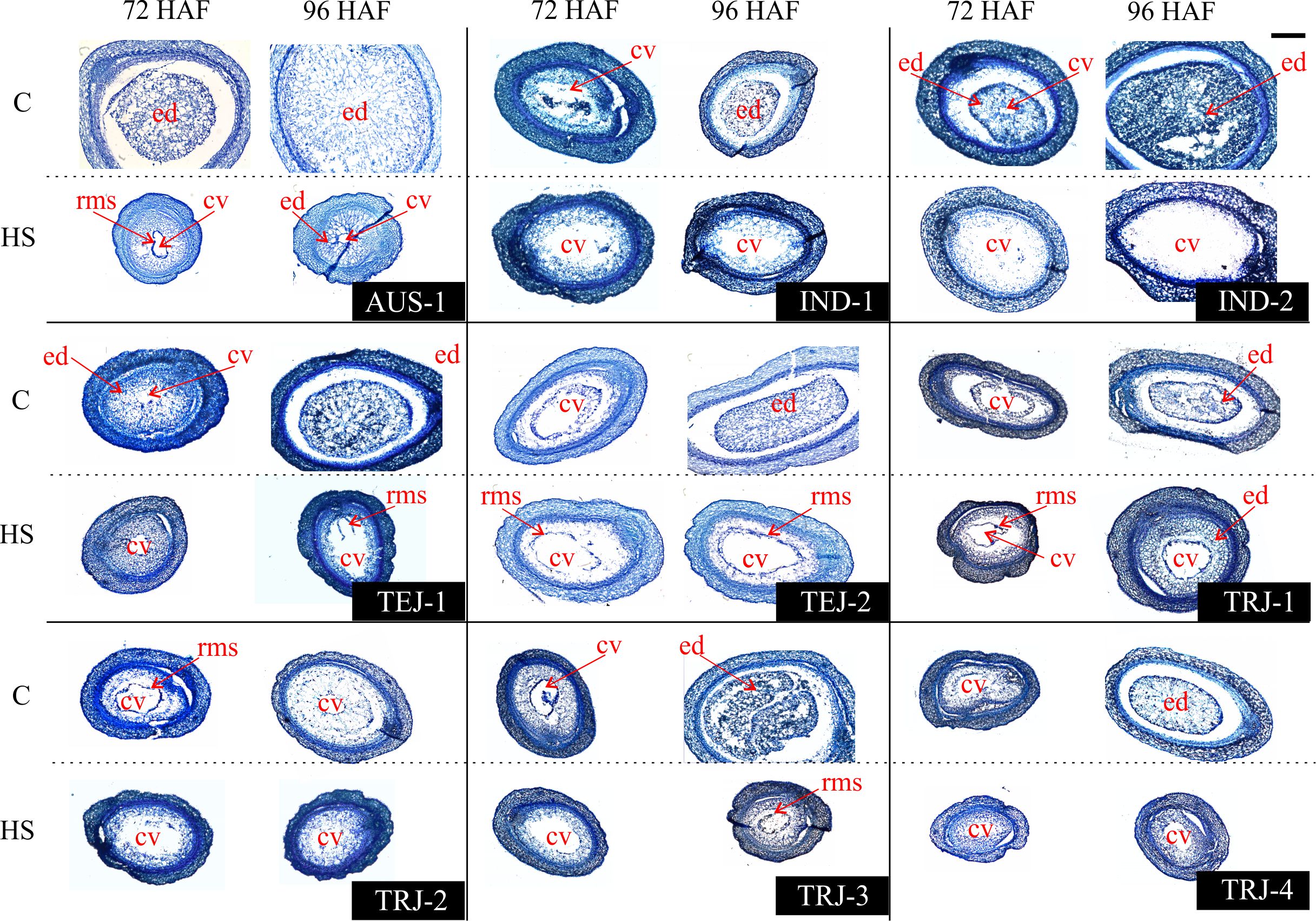

Supplement: Supplementary file 2 [file PLD3-4-e00196-s002.jpg]
